# Timed Rise from Floor as a Predictor of Disease Progression in Duchenne Muscular Dystrophy: An Observational Study

**DOI:** 10.1371/journal.pone.0151445

**Published:** 2016-03-16

**Authors:** Elena S. Mazzone, Giorgia Coratti, Maria Pia Sormani, Sonia Messina, Marika Pane, Adele D'Amico, Giulia Colia, Lavinia Fanelli, Angela Berardinelli, Alice Gardani, Valentina Lanzillotta, Paola D’Ambrosio, Roberta Petillo, Filippo Cavallaro, Silvia Frosini, Luca Bello, Serena Bonfiglio, Roberto De Sanctis, Enrica Rolle, Nicola Forcina, Francesca Magri, Gianluca Vita, Concetta Palermo, Maria Alice Donati, Elena Procopio, Maria Teresa Arnoldi, Giovanni Baranello, Tiziana Mongini, Antonella Pini, Roberta Battini, Elena Pegoraro, Yvan Torrente, Stefano C. Previtali, Claudio Bruno, Luisa Politano, Giacomo P. Comi, Maria Grazia D’Angelo, Enrico Bertini, Eugenio Mercuri

**Affiliations:** 1 Department of Paediatric Neurology, Catholic University, Rome, Italy; 2 Biostatistics Unit, Department of Health Sciences, University of Genoa, Genoa, Italy; 3 Department of Neurosciences and Nemo and Clinical Center, Psychiatry and Anaesthesiology, University of Messina, Messina, Italy; 4 Unit of Neuromuscular and Neurodegenerative Diseases, Department of Neurosciences, Bambino Gesù Children's Hospital, Rome, Italy; 5 Child Neurology and Psychiatry Unit, “C. Mondino” Foundation, Pavia, Italy; 6 Neuromuscular Disease Unit, G. Gaslini Institute, Genoa, Italy; 7 Cardiomiologia e genetica medica, Dipartimento di Medicina Sperimentale, Seconda Università di Napoli, Napoli, Italy; 8 Department of Developmental Neuroscience, Stella Maris Institute, Pisa,Italy; 9 Department of Neurosciences, University of Padua, Padua, Italy; 10 Child Neurology and Psychiatry Unit, IRCCS Istituto delle Scienze Neurologiche di Bologna, Bologna, Italy; 11 Neuromuscular Center, SG. Battista Hospital, University of Turin, Turin, Italy; 12 Dino Ferrari Centre, Neuroscience Section, Department of Pathophysiology and Transplantation (DEPT), University of Milan, Neurology Unit, IRCSS Foudation, Ca’ Granda Ospedale Maggiore Policlinico, Milan, Italy; 13 Metabolic and Neuromuscular Unit, Meyer Hospital, Florence, Italy; 14 Developmental Neurology Unit, Istituto Neurologico “Besta” Milan, Milan, Italy; 15 Neuromuscular repair unit, Inspe and division of neuroscience, IRCSS San Raffaele Scientific Institute, Milan, Italy; 16 Neuromuscular Disorders Unit, Scientific Institute IRCCS E. Medea, 23842, Bosisio Parini, Lecco, Italy; Stem Cell Research Institute, BELGIUM

## Abstract

**Background:**

The role of timed items, and more specifically, of the time to rise from the floor, has been reported as an early prognostic factor for disease progression and loss of ambulation. The aim of our study was to investigate the possible effect of the time to rise from the floor test on the changes observed on the 6MWT over 12 months in a cohort of ambulant Duchenne boys.

**Subjects and methods:**

A total of 487 12-month data points were collected from 215 ambulant Duchenne boys. The age ranged between 5.0 and 20.0 years (mean 8.48 ±2.48 DS).

**Results:**

The results of the time to rise from the floor at baseline ranged from 1.2 to 29.4 seconds in the boys who could perform the test. 49 patients were unable to perform the test at baseline and 87 at 12 month The 6MWT values ranged from 82 to 567 meters at baseline. 3 patients lost the ability to perform the 6mwt at 12 months. The correlation between time to rise from the floor and 6MWT at baseline was high (r = 0.6, p<0.01).

**Conclusions:**

Both time to rise from the floor and baseline 6MWT were relevant for predicting 6MWT changes in the group above the age of 7 years, with no interaction between the two measures, as the impact of time to rise from the floor on 6MWT change was similar in the patients below and above 350 m. Our results suggest that, time to rise from the floor can be considered an additional important prognostic factor of 12 month changes on the 6MWT and, more generally, of disease progression.

## Introduction

The advent of clinical trials in Duchenne muscular dystrophy (DMD) has highlighted the need for natural history studies, defining the progression of the disease. Most of the recent or ongoing clinical trials in DMD have focused on ambulatory patients using the 6-minute walk test, a measure of function and endurance, as the primary outcome.

Both placebo data from the first study using the 6 minute walk test (6MWT) and subsequent natural history longitudinal studies highlighted a high variability in changes in the 6 minute walk distance (6MWD), ranging from patients who improve during the course of the trial to others who lose function and, in some cases, the ability to walk independently[1–6]. There was also an effort to identify different profiles of progression and early predictors of loss of ambulation that have been used to define inclusion and exclusion criteria in the more recent and ongoing studies. The results of these studies, from different international groups and networks have demonstrated that some variables, such as age or 6MWT at baseline can help to identify different trajectories of progression, with boys younger than 7 years showing some improvement in their 6MWT over 12 and 24 months as opposed to those above 7 years who show a progressive decline[3,7,8]. Similarly, boys with baseline values above 350 m remain more stable than those with a 6MWD below 350 meters. The combination of the two variables, age and baseline 6MWT, appears to increase the power to predict possible trajectories of progression over 12, 24 and 36 months[1,3,7,8].

It has been suggested that a similar approach, using other variables such as timed items, could be applied to further reduce the high levels of variation observed in DMD disease progression.

The aim of the present study was to investigate, in addition to known variables, such as age and baseline 6MWT, the possible effect of the time to rise from the floor (TRF) on disease progression and, more specifically, on the changes observed on the 6MWT over 12 months.

## Subjects and Methods

The study is a prospective multicentric study involving 13 of the leading tertiary neuromuscular centers in Italy. Patient inclusion criteria was: genetically proven DMD diagnosis, patient still ambulant and able to walk at least 75 meters independently, without any help, no severe or moderate learning difficulties or behavioral problems.

The study was approved by the Ethical Committees of all the participating centers (Catholic University, Rome; University of Messina; Bambino Gesù Hospital, Rome; Mondino Institute, Pavia; Gaslini Institute, Genoa; Besta Institute, Milan; Stella Maris Institute, Pisa; Maggiore Hospital, Bologna; University of Naples; University of Turin; University of Padua; University of Milano, Nemo Clinical Center, Milan). As the assessments were already part of the clinical routine in all centers, with the approval of the Ethics Committees, verbal consent to anonymously record the data in a database was obtained by the parents for the boys under age.

Details of steroids treatment were annotated for each patient. As the various centers used different types of steroids (deflazacort and prednisone) and had different regimes, we broadly subdivided our cohort into: a) no steroids, boys who had never been on steroids or who had used them for less than a year; b) intermittent regime, all patients with alternate days or alternate weeks or 10 days on/10 days off, for at least a year; c) daily regime, patients who had been on daily treatment of .75 mg of prednisolone or .9 mg/kg/day of deflazacort for over a year, also including those in whom the dose had not been always completely adjusted to the current weight. A small number of patients who took deflazacort on alternate days but with a bolus of 2 mg/kg/day were also included in this group as their monthly dose was similar if not higher to those with a daily standard dose of steroids.

As part of this study all centers performed at each visit the timed items, as part of the North Star Ambulatory Assessment (NSAA) assessment, followed by the 6MWT. Details of the interobserver reliability among the participating centers have already been reported in a previous study assessing the suitability of NSAA and 6MWT in a multicentric setting[9].

### Time to rise from the floor

TRF was performed as part of the NSAA, recording the time taken to complete the task.

It measures the time taken to rise from supine to standing. As in children who are unable to perform this task the time cannot be measured, a time equal or bigger than the worst performance in the group is subjectively given to indicate poor performance.

### 6MWT

All centers were asked to perform the functional assessment in all DMD ambulant boys older than 5 attending their ambulatory units. 6MWT was performed according to the modified ATS guidelines[10].

### Statistical analysis

Descriptive statistics of baseline variables and the possible effect of baseline age, 6MWT and TRF on 12 month 6MWT changes was assessed by a mixed effect linear model including interaction terms,. accounting for the repeated measures within subjects. Correlations were evaluated by a Spermann rank correlation coefficient. Predefined cut off points at baseline for TRF (below and above 7 seconds), age (below and above 7 seconds) and 6MWT (below and above 350m) were used to classify groups of subjects according to their baseline values.

## Results

Two hundred fifteen ambulant DMD boys fulfilled the inclusion criteria. A total of 487 12 month data points collected on consecutive patients between 2008 and 2014 were available for analysis. The age ranged between 5.0 and 20.0 years (mean 8.48 ±2.48 DS); 429 of these assessments were performed on patients on steroids, (174 on intermittent and 255 on daily regimen). All the values presented were corrected for the repeated measures within subjects.

### Time to rise from the floor

The TRF ranged from 1.2 to 29.4 seconds. At baseline, 302 assessments had values below 7 seconds, 165 above 7 seconds, 49 of the 165 of the assessment could not be performed because they were unable to rise without external support.

The mean 12 month changes of the 487 assessments was 2.96 sec.

### 6MWT

The 6MWD ranged from 82 to 567 meters at baseline and between 82 and 614 meters at 12 months. The mean 12 month changes of the 487 assessments was -30.49 meters in the whole cohort, 36 patients lost the ability to perform the 6mwt at 12 months.

### TRF and 6MWT Baseline values

**Fig 1:**
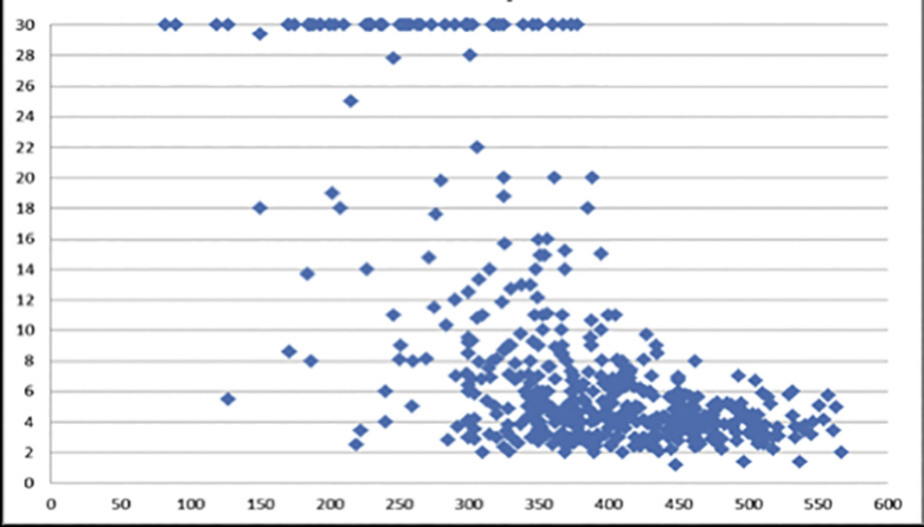
Individual details of TRF scores (in seconds) and 6MWT results (in meters).

When all the assessments in the whole cohort were considered, he mean baseline 6MWD was 413.06 m in the assessments performed in boys with a TRF <7 seconds and 308,20 m in those with a TRF ≥ 7 seconds. Fig 1 shows 6MWT and TRF at baseline.

In the *assessments performed in patients below 7 years of age* (n = 144) the mean baseline 6MWD was 392.18 m in the assessments performed in boys with a TRF within 7 seconds and 322.50 m in those with a TRF in more than 7 seconds.

In the assessments performed in *patients above 7 years of age* (n = 343) the mean baseline 6MWD was 427.08 m in the assessments performed in boys with a TRF within 7 seconds and 307.82 m those with a TRF of more than 7 seconds.

### Baseline TRF and loss of ambulation

**Fig 2:**
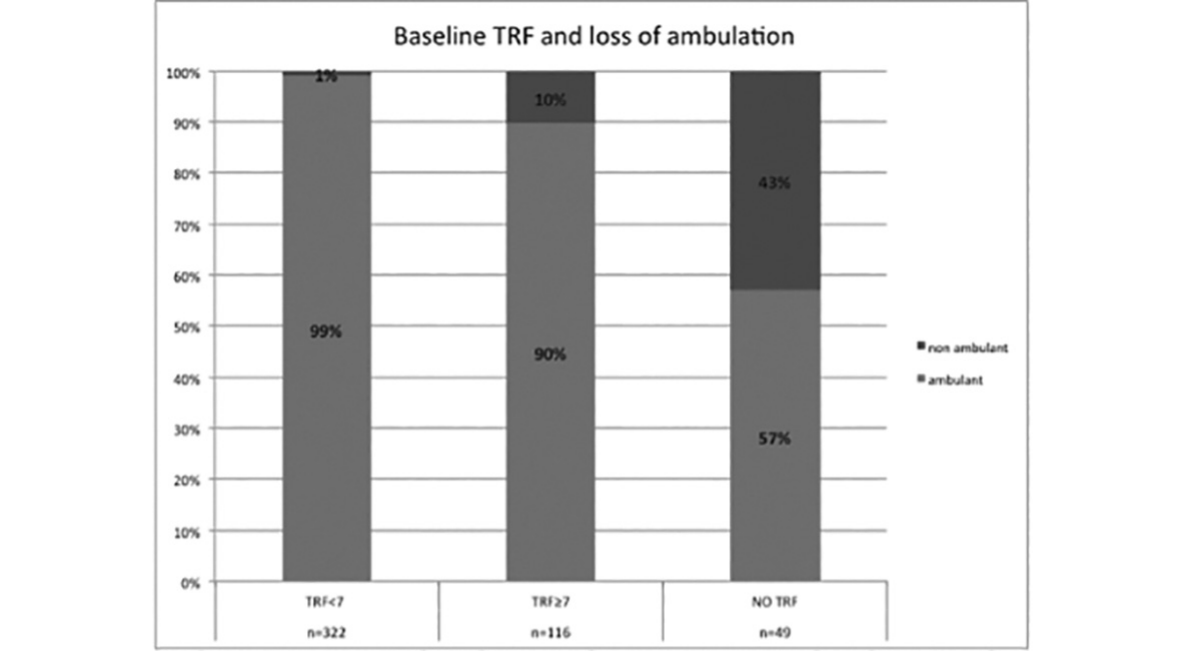
Percentage of patients losing ambulation in subgroups subdivided according to TRF performance.

Thirty-six patients lost ambulation during the 12 month interval. 21 of the 36 were not able to perform TRF at baseline, 12 had baseline TRF above 7 seconds and 3 below 7 seconds. (Fig 2)

### Baseline TRF and 12 month 6MWD changes

The mean 12 month 6MWD change was -30.49 meters in the assessments performed in the whole cohort (n = 487), -6.23 in those with a baseline TRF within 7 seconds (n = 322) and -77.84 in those with more than 7 seconds (n = 165).

In the assessments performed in patients below the age of 7 (n = 144), the mean 6MWD changes were 12.73 in the 130 assessments performed in boys with a TRF within 7 sec and -8.61 in those with a TRF in more than 7 seconds (n = 14).

In the assessments performed in patients above the age of 7, the mean 6MWD changes were -19.07 in the 192 assessments performed in boys with a TRF within 7 sec and -84.26 in those with a TRF of more than 7 seconds (n = 151).

**Fig 3:**
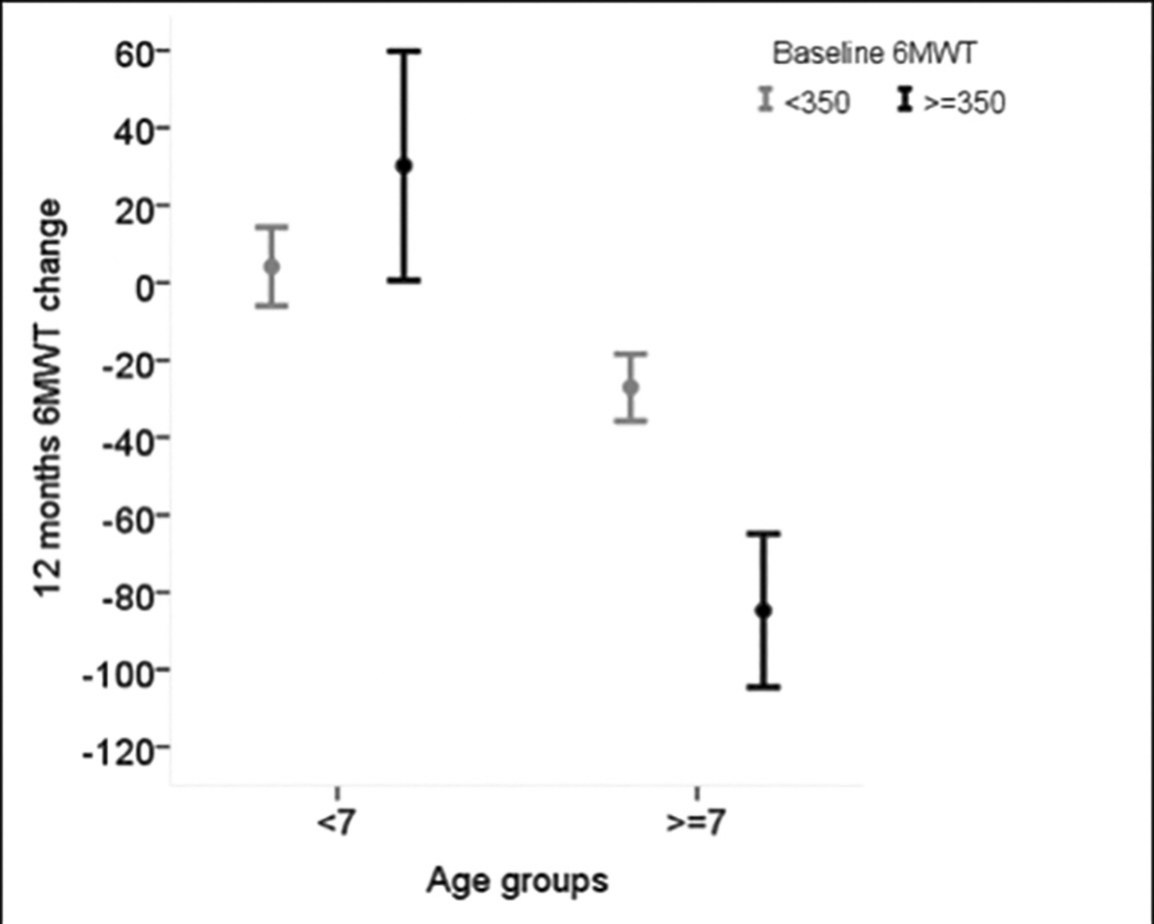
Average 6MWD change over 12 months according to baseline age (<7 years vs > = 7 years) in those with baseline 6MWD< 350 m and in those with baseline 6MWD> = 350 m. The significant age by 6MWD interaction (p<0.001) indicates that the baseline value of 6MWD has an impact on the 6MWD change only in children older than 7 years.

In the whole cohort, 6MWD changes depended on both age and 6MWD baseline value, and a significant interaction (p<0.001) between baseline age and 6MWD was detected: the impact of baseline 6MWT on the 12 month change was relevant only in patients older than 7 years (Fig 3).

**Fig 4:**
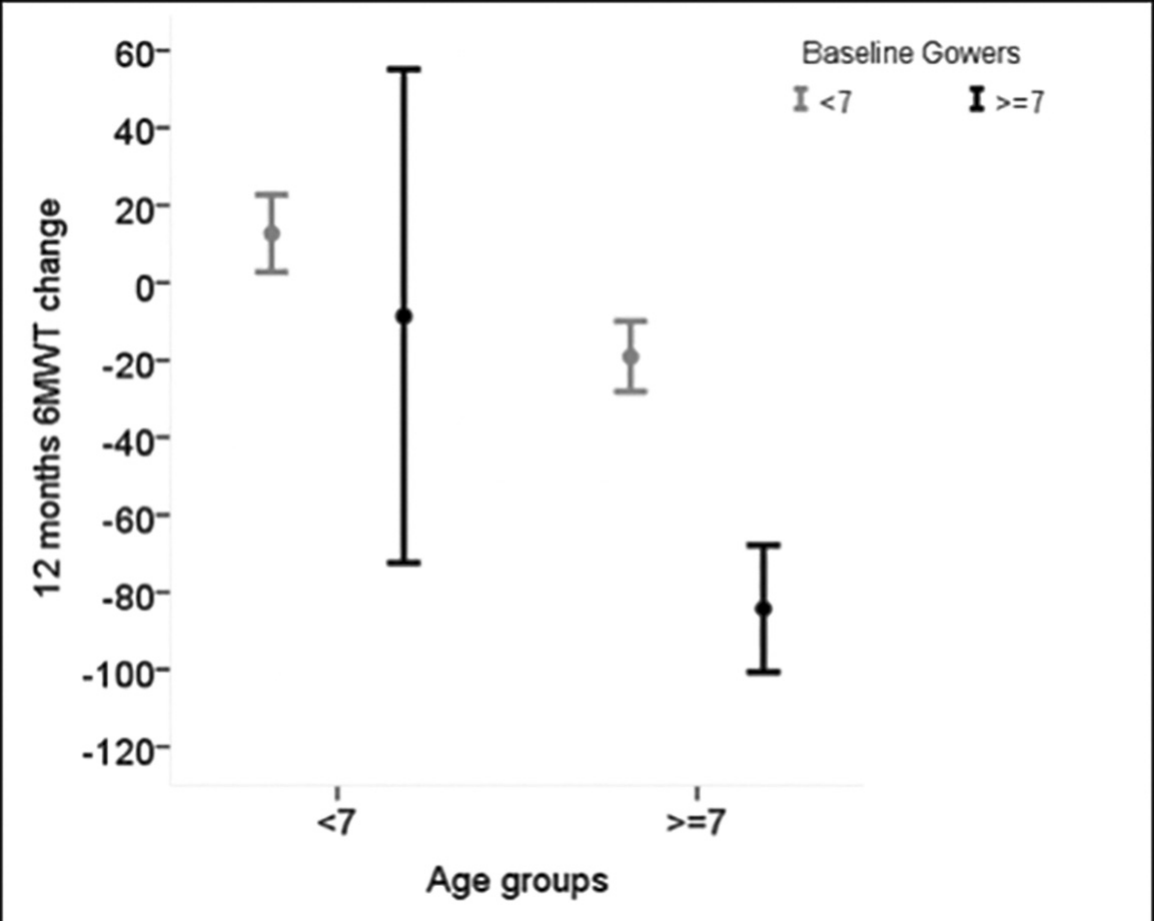
Average 6MWD change over 12 months according to baseline age (<7 years vs > = 7 years) in those with baseline TRF < 7 sec and in those with baseline TRF> = 7 sec. The borderline significant age by TRF interaction (p = 0.06) indicates that the baseline value of TRF has an impact on the 6MWD change only in children older than 7 years.

Similar findings were also observed when evaluating TRF and age: the impact of TRF on the 12 month 6MWD change was relevant only in patients older than 7 years (p for interaction = 0.12) (Fig 4 and data in S1 Fig).

**Fig 5:**
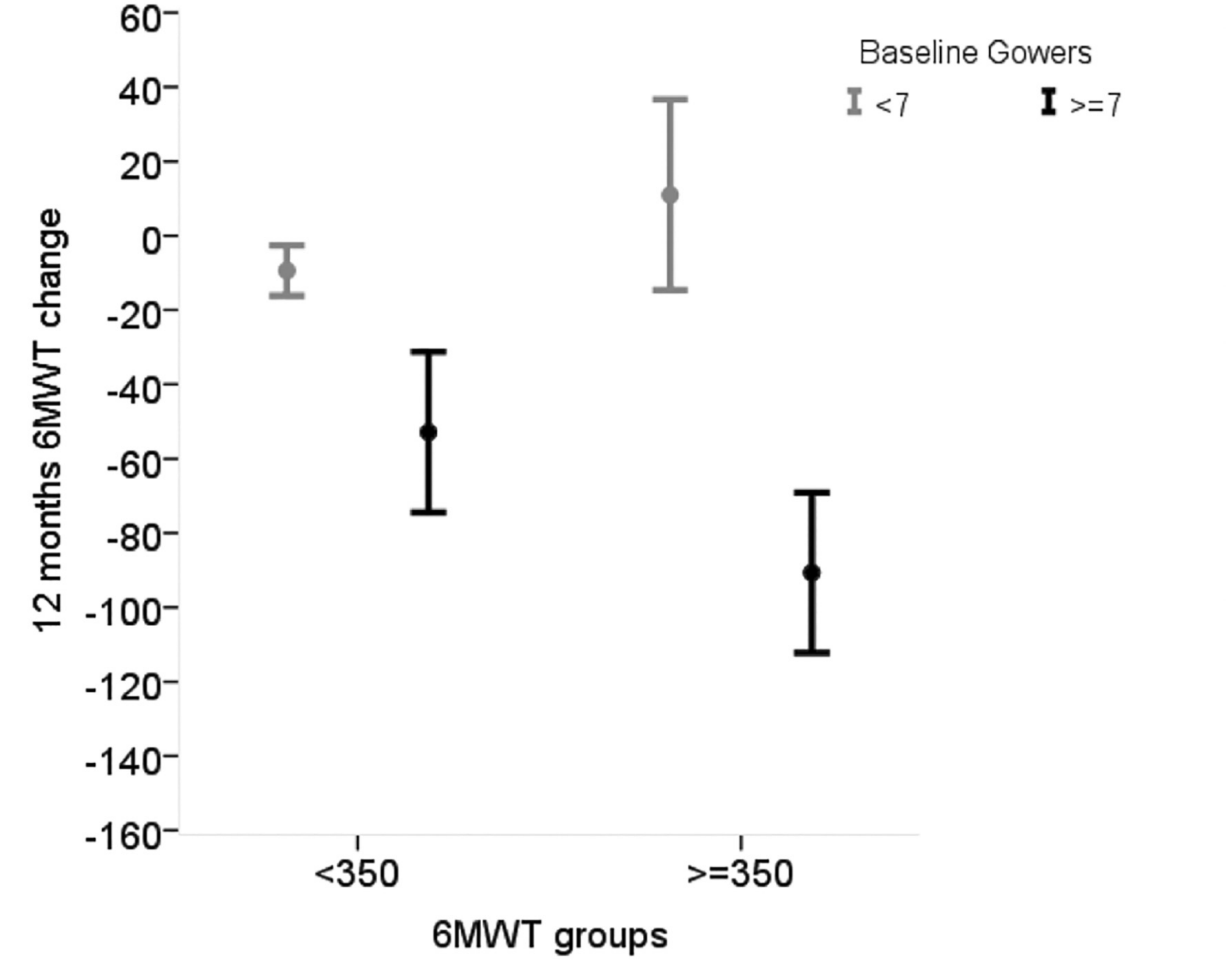
Average 6MWT change over 12 months according to baseline values of 6MWD (<350 vs > = 350) in those with baseline TRF < 7 sec and in those with baseline TRF > = 7sec. There is no 6MWD by TRF interaction, indicating that that the baseline value of 6MWD has the same impact on the 6MWD change according to different baseline TRF values.

In the group above the age of 7 years, both baseline 6MWT and TRF were relevant for predicting 6MWT changes with no interaction between the two measures, as the impact of TRF was similar in the patients below and above 350 m (Fig 5).

## Discussion

The role of timed items, and more specifically, of the TRF, has already been reported as an early prognostic factor for loss of ambulation[11]. Other papers have reported a good correlation between TRF and 6MWT, the functional assessment that is increasingly adopted as the primary outcome measure in several clinical trials and natural history studies in DMD[10,12].

In our cohort the correlation between TRF and 6MWT at baseline was 0.6. While the two measures showed a better correlation in boys at the lower and higher ends of the 6MWT range, there was a wider variability in those with 6MWD between 250 and 400 meters. A number of boys who were still able to perform the 6MWT were unable to rise from the floor without an external support. It was also of interest that, although the inability to perform the TRF was more often associated with poor scores on the 6MWT, this was not always true for some cases who were unable to perform the TRF and had 6MWD over 200 meters. This can be explained by the fact that 6MWT and TRF are dependent on different movement patterns and muscle groups, and that TRF is more influenced by presence of contractures, excessive weight or height.

Following evidence that baseline age and 6MWT can identify trajectories of 12 month changes on the 6MWT[2,3,8], we were also interested to establish the role of TRF, alone or in combination with other known variables, as a prognostic factor of the progression of the disease. We were able to confirm previous observations from our group and others that age and baseline 6MWT values are predictive of 12-month 6MWT changes, and more generally, of disease progression[1–3,8]. In addition, we were able to demonstrate that the TRF is also an important predictor of 6MWT 12-month changes. TRF in isolation was unable to predict changes in the whole cohort. Boys who were unable to perform TRF more frequently had negative 6MWT changes but this did not always hold true for individual cases.

The effect of TRF was different when the whole cohort was subdivided according to known cut off points for age and baseline 6MWT values. In younger patients the effect of TRF was less obvious. This is probably due to the fact that only less than 10% of the younger boys had TRF >7 sec and only 3, who had a global developmental delay, were unable to perform the test.

In boys older than 7 years in contrast, the 6MWT 12-month changes could be predicted by the baseline TRF. In this older cohort the 12-month negative changes were significantly more marked in the boys with a TRF above 7 seconds than in those with a TRF within 7 seconds. The marked difference holds true even if we exclude the boys who were unable to perform the TRF. The difference cannot only be related to differences in steroid treatment as daily steroids were frequently used in both subgroups below and above 7 seconds (58% vs 54%) (S1 Fig).

In this older group, as expected, baseline 6MWT was also relevant for predicting 6MWT changes but there was no interaction between 6MWT and TRF, as the impact of TRF was similar in the patients below and above 350 m.

When we looked at the role of TRF in predicting loss of ambulation within 12 months, we found that, as expected, the risk of losing ambulation was much higher in the group who were unable to perform TRF baseline but this was not always the case as a number of boys (43%) who were unable to perform TRF at baseline were still ambulant 12 month later and, even if in smaller proportion, loss of ambulation was observed in boys who had baseline TRF below 7 seconds (1%) and above 7 seconds (10%).

Our results therefore suggest that, in addition to baseline 6MWT and age, TRF can be considered an important additional prognostic factor of 12 month changes on the 6MWT and, more generally, of disease progression. In boys above the age of 7 years, a poor performance on the TRF significantly increased the risk of marked negative changes on the 6MWT. This information will be of help at the time of designing clinical trials and establishing stratification criteria. Further studies with longer longitudinal data, will help to better establish to which extent the combination of TRF and 6MWT can predict loss of ambulation and disease progression.

## Supporting Information

S1 Fig Details of 6MWT changes in the whole cohort above the age of 7 subdivided according to baseline 6MWT and use of steroids. (TIF)
