# Supplementary figures and images for: Timed Rise from Floor as a Predictor of Disease Progression in Duchenne Muscular Dystrophy: An Observational Study
Source: PLoS One. 2016 Mar 16;11(3):e0151445. doi: 10.1371/journal.pone.0151445 (PMC4794120; doi:10.1371/journal.pone.0151445)

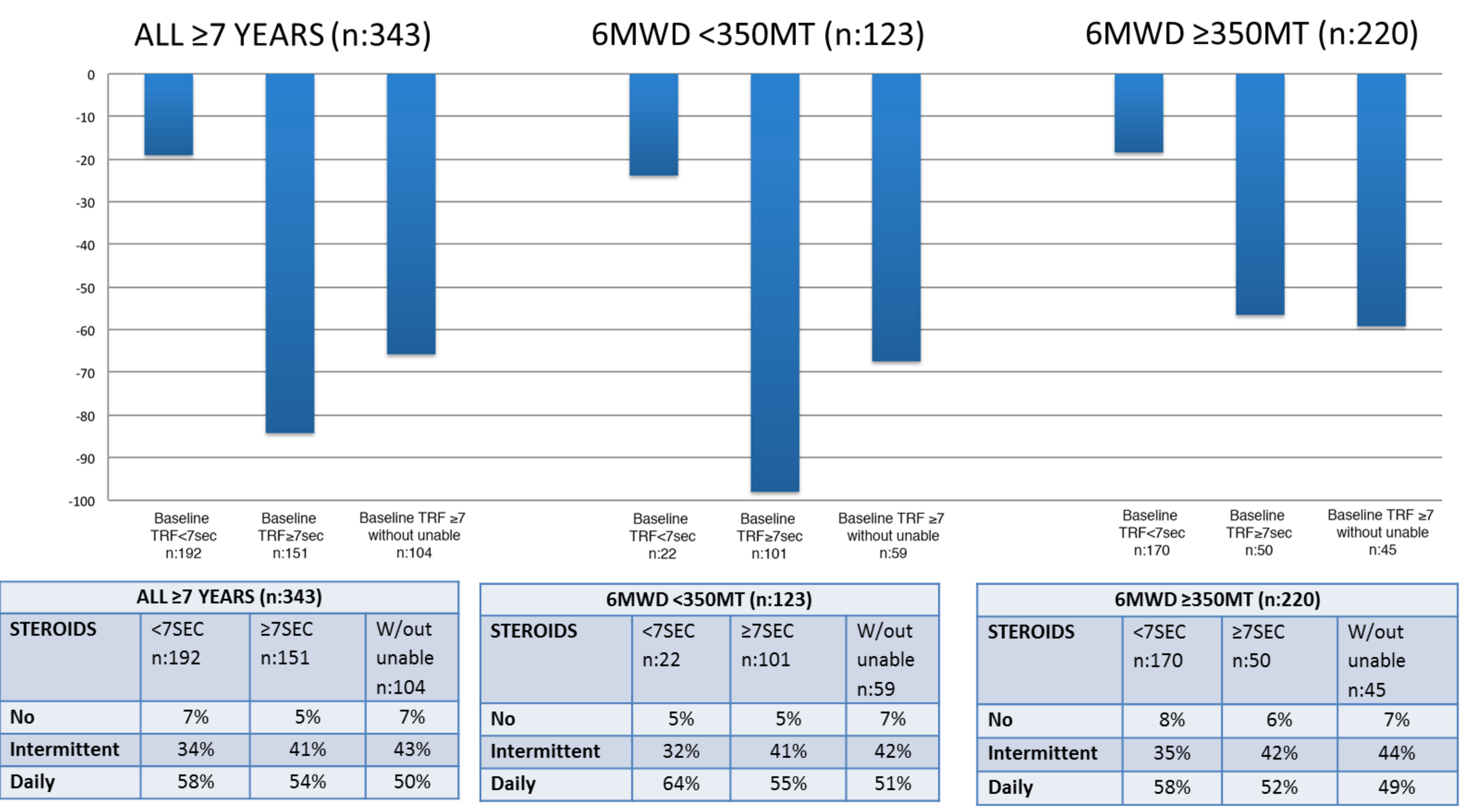

Supplement: S1 Fig — (TIF) [file pone.0151445.s001.TIF]
